# Mental health and disability research in Ghana: a rapid review

**DOI:** 10.11604/pamj.2023.45.166.38808

**Published:** 2023-08-17

**Authors:** Grace Mwangi, Lionel Sakyi, Kenneth Ayuurebobi Ae-Ngibise, Crick Lund, Benedict Weobong

**Affiliations:** 1 Tropical Health, London, United Kingdom,; 2 Ghana Somubi Dwumadie (Ghana Participation Programme), East Legon, Accra, Ghana,; 3 Kintampo Health Research Centre, Research and Development Division, Ghana Health Service, Kintampo North Municipality, Bono East Region, Ghana,; 4 Centre for Global Mental Health, Health Service and Population Research Department, Institute of Psychiatry, Psychology and Neuroscience, King's Global Health Institute, King's College London, London, United Kingdom,; 5 Alan J Flisher Centre for Public Mental Health, Department of Psychiatry and Mental Health, University of Cape Town, Cape Town, South Africa,; 6 Department of Social and Behavioural Sciences, College of Health Sciences, University of Ghana, Accra, Ghana

**Keywords:** Mental health, disability, Ghana

## Abstract

The objective of this rapid review was to explore the current evidence base for mental health and disability research in Ghana. The PRISMA-ScR (Preferred Reporting Items for Systematic reviews and Meta-Analyses extension for Scoping Reviews) checklist was followed. Online databases were used to identify primary studies, systematic reviews, meta-analyses, rapid reviews, or guidelines published between 2010 and 2020. All relevant published (both peer-reviewed articles and grey literature) on mental health and/or disability research conducted in or on Ghana between 2010 and 2020 were included in this review. 4,791 articles were identified in the initial search. After the removal of duplicates, followed by title and abstract screening, 930 articles were selected for full-text review. An additional 8 articles identified from reference lists of included articles were also included in full-text review. After review, 375 articles were selected for inclusion; 234 (62%) were on mental health while the remaining 141 (38%) were on disability. There is an increasing trend in the absolute number of mental health and/or disability studies. Most of the mental health studies included in this review were either observational quantitative studies (n=132; 56%) or observational qualitative studies (n=79; 34%). There were very few interventional studies (n=6; 3%). A similar finding was noted for the disability studies. External funding accounted for 51% of mental health articles. Although there was a steady year-on-year increase in the absolute number of mental health and/or disability studies conducted between 2010 to 2020, there is a need for more intervention studies to evaluate what mental health and/or disability interventions work, for whom, and under what circumstances. These should include evaluations of the cost, benefits, effectiveness, and acceptability of various interventions for policy and planning. Further, there is a need for the Ministry of Health to prioritize research funding for mental health and disability and enhance technical and methodological capacity of researchers to conduct disability and mental health research in Ghana.

## Introduction

The prevalence of mental health conditions and other disabilities has been increasing in low-and-middle-income countries (LMICs) [1]. According to 2019 estimates from the Global Burden of Disease (GBD) [2], approximately 2.6 million people living in Ghana (10.7% of the country's population) have a mental health condition such as schizophrenia, bipolar disorder, or major depressive disorder. Yet only 2% of people living with a mental health condition receive treatment in Ghana [2]. Research is critical to inform policy and practice to narrow this mental health treatment gap, but only 0.38% of the nation's gross domestic product is spent on research and development [1]. Indeed, disparities in mental health research are likely to pose significant challenges in achieving the sustainable development goals [3]. The Government of Ghana passed a Disability Act in 2006 (Act 715) [4] and ratified the Convention on the Rights of Persons with Disabilities in 2012, affirming its recognition of the rights of people with disabilities. Ghana's Mental Health Act (Act 846) was also passed in 2012 [5]. These measures appear to have prompted a surge of research on disability and mental health in Ghana based on the number of studies conducted before the Act and after the Act. A previous review by Read and Doku documented the mental health research landscape in Ghana [6]. Ten years have passed since that review, and subsequent developments such as the expansion of the field of Global Mental Health [7], and Ghana's Mental Health Act 846 which was passed around the time of Read's review, suggest a new review is warranted.

The identification of what currently exists, and any gaps in mental health and disability research can be supported by well-designed review studies. Rapid reviews are particularly useful for providing timely information for decision-making, especially when resources to conduct a full systematic review are limited [8]. Similar to systematic reviews, rapid reviews can influence policy because of the rigorous albeit time-limited approach [8]. However, despite being useful, the utility of a rapid review in determining what currently exists in mental health and disability research in the Ghanaian context is uncertain. Nonetheless, the lessons from this review could be transferable and can inform other African countries who may wish to conduct similar evidence-generating studies, particularly for the neglected areas of mental health and disability research. The aim of this paper was to examine the current landscape of mental health and disability research conducted in or on Ghana in order to synthesize our current knowledge, identify any research gaps and identify avenues for future research. This study differs from the previous review by Read and Doku [6] in two ways: first, its scope includes disability research, which is frequently neglected [9]. Second, it includes data on research funding. This is important to examine funding sources and the relationship between funding and research output [10].

## Methods

### Study design

This study employed the approach of a rapid review. The main reason why we chose this methodology was to narrow the scope of the research question and reduce the extent of data abstraction with the aim to provide evidence in a timely and cost-effective manner. This was in response to a specific requirement of Ghana Somubi Dwumadie to provide a high-quality evidence summary data to help in the selection of demonstration districts for the implementation of mental healthcare plans. As mentioned, rapid reviews are particularly useful for providing timely information for decision-making, especially when resources to conduct a full systematic review are limited [8]. They also offer accelerated knowledge synthesis through a streamlined timely and cost-effective approach that is directed and guided by stakeholders as was the case in this review [11]. Although this was not a scoping review, the PRISMA-ScR checklist was followed to help the researchers develop a greater understanding of relevant terminology, core concepts, and key items to report in this review [12]. Ethical approval for the study was obtained from the Ghana Health Service Ethics Review Committee (GHS-ERC025/08/20) and King's College London Research Ethics Committee (LRS-20/21-20,866). The protocol for this study was reviewed by two ethics committees but was neither registered nor published.

### Search

This rapid review was conducted in November 2020. A wide range of terms and definitions have been used to define mental health, with different and partly overlapping meanings. The World Health Organization (WHO) defines mental health as “a state of well-being in which the individual realizes his or her own abilities, can cope with the normal stresses of life, can work productively and fruitfully, and is able to make a contribution to his or her community” [13]. It is more than simply the absence of mental health conditions. The International Classification of Functioning, Disability and Health (ICF), on the other hand, defines disability as an umbrella term for impairments, activity limitations and participation restrictions. Disability is the interaction between individuals with a health condition (for example cerebral palsy) and personal and environmental factors (for example negative attitudes, inaccessible facilities, and limited social supports) [14]. For this review, the above definitions were adopted in the screening of research articles for inclusion. A comprehensive search of electronic databases (PubMed, Embase, PsycINFO and Scopus) was done to identify relevant studies on mental health and/or disability conducted in or on Ghana over the period The Cochrane database was also searched to identify any relevant systematic reviews conducted on mental health and/or disability in Ghana. Reference lists of selected articles and relevant systematic reviews were screened to identify any other relevant articles that were missed during the initial database search. Both peer-reviewed articles and grey literature were included in this review regardless of the study design and the study population. The use of grey literature has been highlighted in the context of Mental Health studies in LMICs as a key to acknowledge the research/dissemination realities of many LMICs, but also to generate findings that reinforce and/or expand those documented in peer-reviewed articles [15].

#### Inclusion and exclusion criteria

All published peer-reviewed articles and grey literature papers on mental health and/or disability research conducted in or on all 16 regions of Ghana in the 10-year period (2010 to 2020) were included in this review. Prior to 2018 when six new regions were created, Ghana had 10 regions (Greater Accra, Ashanti, Central, Western, Volta, Eastern, Brong-Ahafo, Northern, Upper East, Upper West). Four of these 'traditional' regions were split to create six new regions. Western region was split to create Western-north; Volta region created Oti region; Brong-Ahafo created Bono East and Bono regions; Northern created Savannah and North-East regions. All types of studies were included regardless of the study design and the study population. However, due to time limitations, only articles published in English were included.

#### Key search terms

The search strategy for electronic databases incorporated both medical subject headings (MeSH terms) and free-text key terms adapted to suit each individual database using applicable controlled vocabulary. The key search terms used in this review are ((“mental health”[All Fields] OR (“mental health” [MeSH Terms] OR (“mental” [All Fields] AND “health” [All Fields]) OR “mental health” [All Fields])) OR disability [All Fields]) AND (“ghana” [MeSH Terms] OR “ghana” All Fields]). The full search strategy is provided in the appendices.

### Selection of sources of evidence

Multiple sources of evidence were targeted for this review. These included reviews on quantitative and qualitative research, expert opinion, and policy documents. Screening and eligibility processes are as described above.

### Data charting

For the articles included in this review, data was extracted using a standardized template that included study title, first author, year of publication, literature type, study design, study population and setting, research funding and research implementers. In addition, research topics were identified through thematic analysis and organized into themes. In this case, the researchers read through all the included papers and identified patterns in the findings across the papers to derive themes.

## Current status of knowledge

### Results

**Sources of evidence**

**Figure 1:**
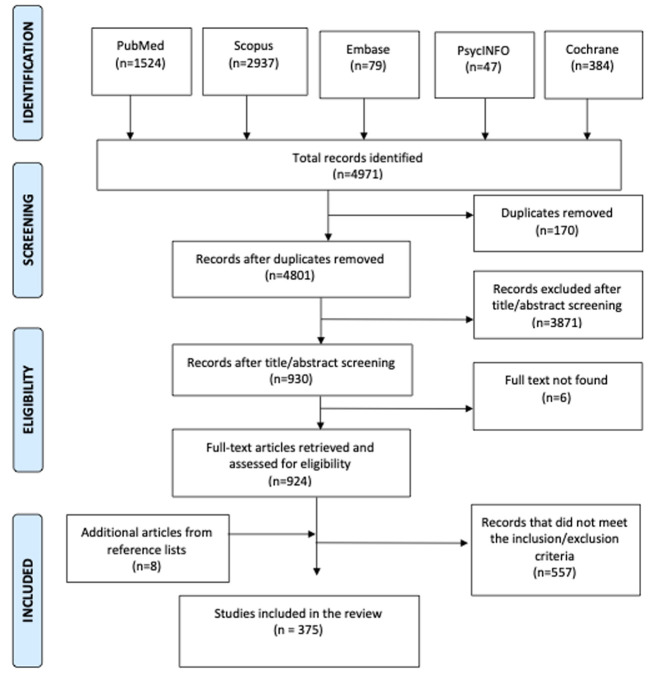
PRISMA-ScR flow chart of article selection

Figure 1 shows the article selection process. The initial search yielded 4,791 articles. After removal of duplicates, followed by title and abstract screening, 930 articles were selected for full-text review. An additional 8 articles identified from reference lists of included articles were also included in full-text review. After review, 375 articles were selected for inclusion. Editorial articles, reviews, expert opinion pieces, conference papers and meeting abstracts were excluded, as were studies that were not conducted in or on Ghana and those whose focus was not mental health and/or disability.

#### *Distribution of data retrieved and demographics of study populations*.

Of the 375 articles included in this review, 234 (62%) were on mental health while the remaining 141 (38%) were on disability. The study population for the mental health studies consisted of different age groups ranging from children under 17 years to adults aged 50 years and above. 23 studies (10%) were conducted among participants in the 50+ age group, while another 20 studies (9%) were in among the 18+ age group. There were few studies conducted among adolescents (n=6; 3%) and children (n=6; 2%). In terms of gender disaggregation, 5 of the 234 mental health articles (2%) were from studies conducted among men only. The remaining 29 articles (12%) were from studies conducted on women only, while a majority (n=198; 85%) included both men and women. For the disability studies, study samples included: people with disabilities (48 studies; 34%); caregivers (25 studies; 18%); general population (28 studies; 20%); and health care professionals (5 studies; 4%). Others included traditional and faith-based healers (n=1; 1%), and mixed population groups (8 studies; 6%). In terms of age, majority of the disability articles (13%; n=19) were from studies conducted in the 50+ age group, especially those drawn from the WHO Study on Global AGEing and Adult Health (SAGE). There were very few studies on adolescents (n=1) and no studies conducted among children. In terms of gender, 130 (92%) of the studies were designed to include both male and female participants, 13 articles (9%) articles had women only study participants, and no study was focused on men only. The women-only studies were mainly aimed at exploring the role of women as caregivers as well as the experiences and challenges faced by women with disabilities.

#### Research output

**Figure 2:**
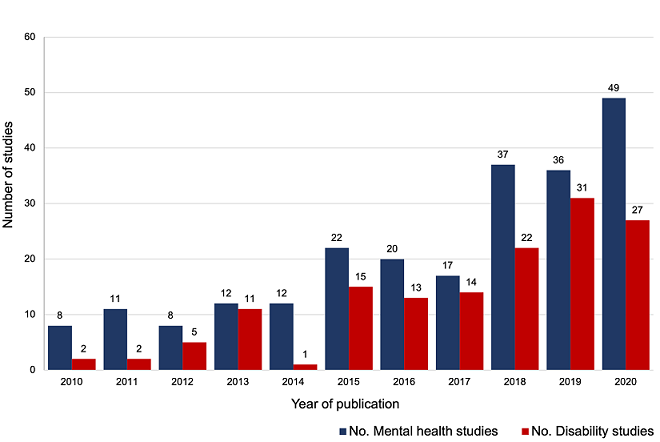
number of mental health and disability studies conducted over the past 10 years (2010-2020)

There has been a steady year on year increase in the absolute number of studies conducted on mental health in Ghana over the 10-year period 2010 to 2020, and majority (98%) of these were peer-reviewed (Figure 2). Three of the 4 grey literature reports were doctoral dissertations, while 1 was a scholarly paper published in a non-indexed university journal. Similarly, there has been a steady increase in the absolute number of disability studies conducted in Ghana over the same period (2010 to 2020) with a notable increase recorded between 2016 and 2019. Worth noting, only one article included in this review was published in 2014 [16]. This shows a sharp decline in articles published that year compared to the other years. However, just from the review, we could not tell the reasons behind this decline.

### Study designs

Table 1 shows the study designs employed. Most of the mental health articles included in this review were either from observational quantitative studies (n=132; 56%) or observational qualitative studies (n=79; 34%). There were very few interventional studies (n=6; 2.5%), mixed method studies (n=4; 1.7%) and case studies (n=3; 1.3%). “Other” study designs (n=10) found in this review include ethnographic research (n=5), secondary data analysis (n=2), and policy evaluation, critical discourse analysis and ethnobotanical approach which had one study each. A good proportion of the disability articles included in this review were from observational quantitative studies (n=68; 50%) followed by observational qualitative studies (n=58; 42%). There were a few studies which had adopted mixed- method, case study or other study designs (Table 2). The “other” category includes pre- and post-impact evaluation, secondary data analysis, iterative design approach, critical discourse analysis and photovoice study design. There were no interventional studies focusing on disability found during this review.

**Table 1:** research designs used in mental health studies

Design	Count	% (out of 234)
Observational - quantitative studies	132	56%
Observational - qualitative studies	79	34%
Intervention studies	6	3%
Mixed method studies	4	2%
Case studies	3	1%
Other*	10	4%

* The “other” category includes ethnographic research, secondary data analysis, policy evaluation, critical discourse analysis and ethnobotanical approach which had one study each.

**Table 2:** research designs used in disability studies

Design	Count	% (out of 141)
Observational -quantitative studies	68	48%
Observational - qualitative studies	58	41%
Intervention studies	0	0%
Mixed method study studies	5	4%
Case study studies	5	4%
Other*	5	4%

* The “other” category includes pre- and post-impact evaluation, secondary data analysis, iterative design approach, critical discourse analysis and photovoice study design. There were no interventional studies focusing on disability found during this review

#### Research themes

Of the 234 mental health articles, 55 (24%) articles were on mental health systems in the areas of mental health policy and legislation, health workforce issues and mental health services. For those articles that were mental illness-related, 41 articles (18%) were on general depression and anxiety disorders among adults followed by unspecified mental illness (n=35; 15%), psychological distress (n=33; 14%) pre and postpartum depression (n=23; 9.9%) and schizophrenia or psychosis (n=22, 9%). The least researched mental illnesses were dementia, post-traumatic stress disorder, attention deficit hyperactivity disorder and substance abuse disorders which had one paper each. Seventy-eight (33%) of the 234 mental health-related articles were on prevalence (burden or distribution) and risk factors (determinants or predictors) of mental health-related illness and events (not just mental health conditions) in specified populations within Ghana. Other notable research themes identified include knowledge, attitude, beliefs and practices of patients, caregivers, healthcare providers and society in general (n=48) and social determinants of health (n=35). Issues of caregiving, traditional and faith healing, and the need for collaboration between formal mental health and alternative medicine therapies have also been well researched. Only 4 articles included in this review were on mental health workforce, with a focus on community based mental health workers.

In addition to the key research themes, there were eight cross-cutting themes identified in some of the papers included in this review. These include maternal mental health (n=23; 10%), social capital and mental health (n=20; 9%), stigma and discrimination (n=12; 5%), healthcare disparity (n=6; 3%), policy and legislation (n=5; 2%), gender (n=4; 2%), poverty and human rights (n=3; 1%). For the disability studies, 100 of the 141 articles (71%) were focused on a specific type of disability. Out of these, 15 articles (15%) were on visual impairment followed by multiple disabilities (n=12; 12%). Other types of disabilities which were found in the articles included in this review (not captured in the top 10 disabilities) include sickle cell disease (n=3), leprosy-related disability (n=3), stroke-related disability (n=2), communication disabilities (n=2), Downs Syndrome (n=1), Parkinson's disease (n=1), dyslexia (n=1), and dysphonia (n=1). 45 of the 141 articles (32%) were drawn from epidemiological studies which were aimed at establishing the burden and association between difference risk factors and disability. An additional 28 articles (20%) were focused on knowledge, attitudes, and practices of different population groups towards disability; with 11 of the 28 articles (39%) addressing the issue of stigma and discrimination. No research was found to address implementation of policies and rights of people with disabilities as stipulated in the 2006 Ghana's Disability Act 715 [17].

#### Funding

**Figure 3:**
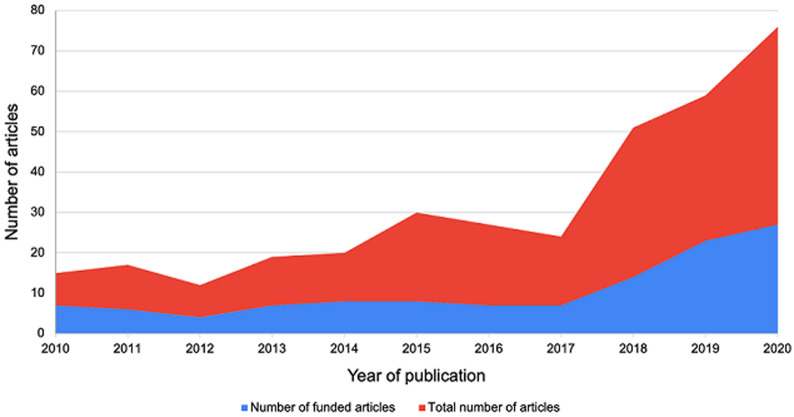
trends in funding and number of mental health articles published between 2010 and 2020

**Figure 4:**
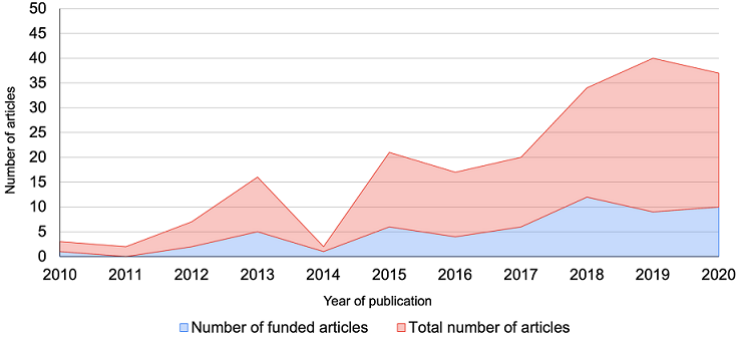
trends in funding and number of disability articles published between 2010 and 2020

118 of the 234 (51%) mental health articles were from studies that had received external funding support. For the remaining 114 articles (49%), 36 articles (32%) were from studies that indicated that they did not receive any external financial support while 78 articles (68%) did not specify whether the study had received any funding (Figure 3). Some of the key funders noted in this review include National Institutes of Health (NIH) (including but not limited to National Institute of Mental Health, National Institute of Neurological Disorder and Stroke, National Institute of Child Health and Development among others) (22 studies; 19%), UK Department for International Development (DfID) (now known as Foreign Commonwealth Development Office (FCDO) (17 studies; 14%), Office of Research Innovations and Development (ORID) of the University of Ghana (7 studies; 6%) and National Research Foundation (NRF) of South Africa (5 studies; 4%) among others. For the disability studies, 56 of the 141 articles (40%) were from studies that had received external funding support. For the remaining 85 articles (60%), 24 (28%) were unfunded and the remaining 63 articles (78%) did not specify whether the study was funded. Some of the notable funders noted in this review include National Institutes of Health (12 studies; 21%), Christian Blind Mission (4 studies; 7%) and Office of Research Innovations and Development (ORID) of the University of Ghana (4 studies; 7%) among others. Figure 4 shows that the number of studies conducted over the period of this review increased as the number of funded studies increased.

#### Implementing institutions

Implementing institutions in this review were defined as either one of the Ghanaian institutions involved in conducting the study (not necessarily the institution affiliated to the first author) or the affiliate institution of the first author (where no Ghanaian institution was involved). Based on this definition, 75% (n=175) of the 234 mental health articles in this review had at least one Ghanaian institution involved in the study, while the remaining 59 articles (25%) were from studies conducted by external institutions (i.e., no Ghanaian institution was associated with any of the authors). For the disability studies, 99/141 (69%) articles had at least one researcher from a Ghanaian institution, while the remaining 31% (42/141) were conducted by external researchers/research institutions.

#### Geographical distribution of studies in Ghana

**Figure 5:**
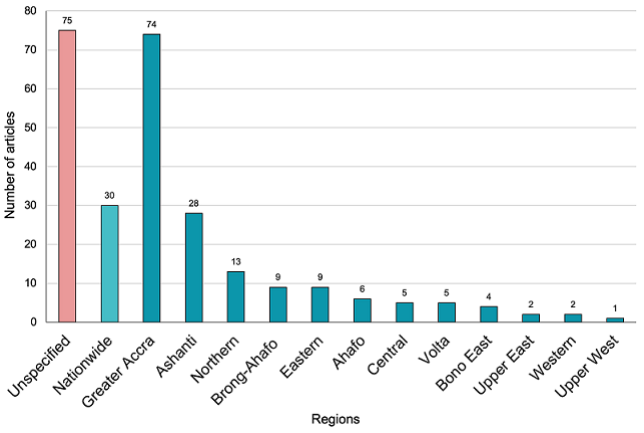
number of mental health articles published by region

All the studies were conducted in Ghana, with 54 mental health studies (23%) and 27 disability studies (19%) being multi-country studies which included Ghana. The multi-country studies included the WHO Study on global AGEing and adult health (SAGE) [18] conducted in China, Ghana, India, Mexico, Russian Federation and South Africa; the Global Kids Online project [19] conducted in Bulgaria, Chile, Ghana, and Philippines; and the Mental Health and Poverty Project (MHaPP) [20] conducted in Ghana, South Africa, Uganda, and Zambia. Figure 5 shows mental health study sites by region. Majority (n=135, 58%) of the mental health studies were conducted only in the southern regions of Ghana, with a small number (n=16, 7%) reported only from the northern sector. Some articles did not specify the region where the studies were conducted (n=74, 32%), while others (n=30, 13%) were from studies that had a nationally representative sample of participants (i.e., covered all regions) such as SAGE. Within the period of the review, some studies were conducted in the new regions created under Brong-Ahafo (6 in Ahafo; 4 in Bono East).

**Figure 6:**
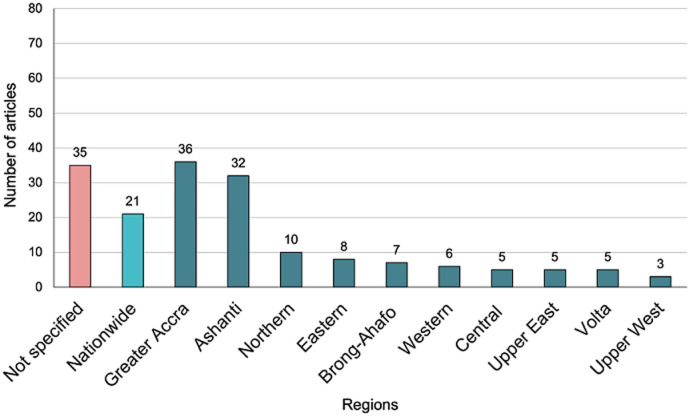
number of disability articles published by region

Figure 6 shows disability study sites by region. Like the mental health research output, majority (n=100, 70%) of the studies were conducted only in the southern regions of Ghana (excluding Ahafo and Bono East regions where no studies were identified), with a small number (n=18, 13%) reported only from the northern sector. 36/141 (25%) articles did not specify the study region, while 21 articles (15%) were from studies that covered all regions. Unlike the mental health studies, all the disability studies recorded in this review were conducted before 2018 in the era of the 10 traditional regions.

## Discussion

This rapid review was conducted to synthesize the current evidence on mental health and disability research activities in or on Ghana over a 10-year period between 2010 and 2020, in order to identify any research gaps and implications for future research. Overall, the review points to an increasing trend in both mental health and disability research output. The review also highlights the fact that one-quarter of the research studies were conducted without the collaboration of Ghanaian institutions, employed observational study designs, were concentrated in the southern regions of Ghana, and the research funding support has been limited and not commensurate with the research output over the period of the review.

Our findings in terms of the increasing trends in mental health and disability research output are consistent with the observed increases in mental health and disability research output globally [21]. The advent of Global Mental Health [22] and the United Nations Convention on the Rights of Persons with Disabilities (2006) [23] appears to have sparked a surge in research into mental health and disability rights globally and Ghana has been no exception. The impact of the field of Global Mental Health has been enormous and is linked to increased investments in mental health research in low and middle-income settings [22]. Similarly, it is possible that the Mental Health Act 846 passed in 2012 may have contributed to an increase in mental health research in Ghana by raising the profile of mental health and disabilities in the country [5].

Whilst our review points to an increase in the absolute numbers of mental health and disability research studies in Ghana, this should be interpreted with caution. As reported elsewhere, when looked at as a proportion of total health-related research publications in Ghana, the trend suggests a decline in the number of mental health and disability reported studies [24]. Based on our review, the role of funding could partly explain this observation. There has not been a substantial increase in the amount of funding over the same period relative to the total volume of research. This is not entirely surprising given Ghana spends less than 1% of the country's gross domestic product on research and development [25]. The technical capacity to design rigorous studies is an important determinant of research output and this may be a challenge in Ghana. The technical capacity to conduct mental health and disability research was rated as weak in a recent study on setting research priorities in Ghana [24].

Aside from the observation that peer-reviewed mental health and disability research output relative to the amount of health research may not have increased since 2010, our review also identified some challenges with the type and approach to the research conducted in Ghana. Overall, it was noted that most of the studies conducted in both mental health and/or disability are observational quantitative or qualitative studies, with very few intervention studies. Read's literature review on mental health research in Ghana in 2012 [6] reported similar observations, suggesting the trend has also not improved since 2010. Although observational studies are useful in establishing the burden of disease and inform possible prevention measures, they do not provide data on the effectiveness or efficacy of interventions. Inadequate funding and technical capacity could explain this observation, as conducting intervention studies is expensive and requires training in advanced epidemiology and trial design [26].

Our review also highlights important imbalances within the country. Majority of the research on mental health and/or disability is concentrated in the southern regions of Ghana. This observation aligns with the skewed distribution of specialist mental health services in Ghana wherein all the three psychiatric hospitals are located in the southern sector, with none in the northern sector [27]. The northern regions of Ghana are among the poorest and likely to present increased risk of mental health conditions; the social determinants theory of mental health conditions supports this assertion [27]. Arguably, these are the settings that will be benefit from well-designed epidemiological and intervention research. Despite this unequal distribution of studies across Ghana, the studies reviewed showed a reasonably equal representation of study participants in terms of age, gender, educational background, profession, income, and socioeconomic status.

As noted by other scholars, research-generated information helps establish the health needs in a given setting [28], provides evidence on culturally appropriate and cost-effective individual and collective interventions [29], including their implementation [30], and very importantly explores the obstacles that prevent recommended strategies from being implemented [31]. Ghana may not be benefiting from these advantages of research as our review clearly highlights the 'research gap' [32] in Ghana.

Our findings have several implications for the way forward. First, the observation that peer-reviewed mental health and disability research output may not have significantly improved since 2010 means there must be a deliberate effort to promote more research on mental health and disability in Ghana. Second, efforts should be made to improve and/or build research capacity nested within a supportive research culture. The weak capacity to conduct high-quality research in LMICs has been previously reported [24,33].

Third, for a period of 10 years, there has been very little increase in the amount of funding available for mental health and/or disability research in Ghana. Even the little funding available came from external funders. We also observe in our review that the least researched mental health conditions were dementia, post-traumatic stress disorder and substance abuse disorders; ageing-related mental health conditions and substance use disorders are becoming common in LMICs and this requires urgent attention [34]. Finally, the fact that one quarter of all research within the period of this review was conducted without the involvement of a Ghanaian institution hints of the undermining effects of colonialist structures that have too long been part of global health research. This requires urgent action and as argued elsewhere, to improve on the past in global mental health, institutions in high-income countries (HICs) must champion equity, solidarity, and true partnerships between people with different experiences, knowledge, and needs from around the world [35].

Recommended strategies by Thornicroft and colleagues [32] to address these challenges are worth mentioning: training mental health professionals in research methods and scientific writing; making mental health research attractive to young researchers; promoting strategies for acquiring research grants and for developing and sustaining researchers' careers; increasing the level of networking among research teams; enhancing research dissemination; and fostering dialogue between research teams and policymakers. Building this capacity will ensure an increase in the profile of well-designed intervention studies to help address critical questions on epidemiology and culturally appropriate interventions that work in our setting. This could be done by increasing the financial capacity of the three Ghana Health Service health research centers, strategically located across the three main ecological belts of Ghana viz. Savanah, Middle, and Southern. This will also help to address the uneven mental health and disability research activity in Ghana.

Aside from the research centers, universities, particularly schools of public health could take up interest and convene formal training programmes and courses in mental health and disability research. To address the issue of funding in mental health and disability research, there is need for more local funding of mental health and disability research. One way to address this is for the Ministry of health to prioritize research funding and implement Section 81, 2 d of Mental Health Act 846 [5]. Other strategies for generating funds for research include South-South or North-South partnerships [36].

Our rapid review has some strengths, including the fact that the approach is useful for providing rigorous and timely information for decision-making, particularly when resources to conduct a full systematic review are limited [37]. Further, our approach to review both mental health and disability research together is novel in our setting. The wide scope also allowed us to include all peer-review studies conducted on mental health and/or disability in Ghana. These notwithstanding, we recognize some limitations. First, due to time constraints, specific mental health conditions and disabilities (e.g., schizophrenia) were not included in the search terms and grey databases were not searched. It is therefore possible that including more search terms and grey literature databases would have produced additional relevant material. Despite this, the search retrieved a substantial number of studies which was adequate to meet the aims of the research and contained a good range of citations written from different perspectives and over a ten-year period. Second, we were unable to assess the rigor or quality of studies included.

## Conclusion

Our synthesis of mental health and disability research in Ghana suggests the current research landscape has not improved; only a modest increase in research output, funding is inadequate, most of the research is concentrated in the southern sector, there is a clear lack of collaboration with local institutions, and use of observational studies dominate research activities. There is a need for more intervention studies to evaluate what mental health and/or disability interventions work, for whom, and under what circumstances. These should include evaluations of the cost, benefits, effectiveness, and acceptability of various interventions for policy and planning. Further, there is a need for the Ministry of Health to prioritize research funding for mental health and disability and enhance technical and methodological capacity of researchers to conduct disability and mental health research in Ghana.

### What is known about this topic

- *Mental health has been a neglected area for research in many countries, and Ghana is no exception;*
- *In Ghana there is limited research in mental health, and most studies employ small sample sizes, thus largely speculative in arriving at conclusions*.
- *Most of the studies conducted in both mental health and/or disability are observational quantitative or qualitative studies, with very few intervention studies*.

### What this study adds

- *We now know the state of disability-related research in Ghana; previous reviews were limited to only mental health research;*
- *The utility of a rapid review in determining what currently exists in mental health and disability research;*
- *Research activity within country is not equitably distributed; majority of the research on mental health and/or disability is concentrated in the southern regions of Ghana*.
